# Advances in the biology of cerebral cavernous malformations

**DOI:** 10.4103/2152-7806.70955

**Published:** 2010-10-11

**Authors:** Juri Kivelev, Aki Laakso, Mika Niemelä, Juha Hernesniemi

**Affiliations:** Department of Neurosurgery, Helsinki University Central Hospital, Topeliuksenkatu 5, 00260 Helsinki, Finland

In this review, the authors have summarized recent investigations on the biology of the cavernous malformations. During the last decades, our understanding of the molecular profile of this pathology has significantly improved. Ultrastructural and immunological changes have been investigated as possible mechanisms of cavernoma evolution. Identification of genes responsible for cavernoma development will likely open new avenues for alternative therapeutic approaches in the future, especially in families affected by hereditary forms of the disease. The complexity of factors having an influence on cavernoma development is, however, evident. Thus, intrinsic molecular perturbations in these lesions must also be appreciated as interacting with extralesional factors, like venous blood flow disturbances in the affected region or intraparenchymal changes in the surrounding brain. A systematic approach, illustrating this pathology as a pattern of multiple changes inside and outside the lesion, seems to be rational for better understanding of the disease. Although a thorough analysis of the accumulating data from laboratory investigations of cavernomas most probably will influence our approach to cavernoma treatment in the near future, many important questions remain open. In our opinion, one of the most intriguing issues is the mechanism of hemorrhage. Although natural history studies have demonstrated relatively low bleeding risks, in certain cases, hemorrhage may cause devastating neurological consequences, indicating active treatment. Traditionally, cavernoma hemorrhages are divided into micro- and macrohemorrhages. Microhemorrhages, leading to accumulation of hemosiderin in the adjacent brain, are well described and related to the fragility of the cavernoma wall. Seemingly, these do not cause stroke-like symptoms, even though they may explain the epileptogenic nature of many cavernomas. The so-called gross or extralesional macrohemorrhages are usually considered as true cavernoma hemorrhages, and the fact that they exist demonstrates the real potential for bleeding in these low-flow vascular malformations. However, analyzing the literature, we have not been able to find any convincing hypothesis of the mechanism of such “gross” hemorrhage, which would be based on hemodynamic principles. A blood flow inside the cavernoma is considered very slow (“angiographically occult lesions”), and no feeding arteries or draining veins can be visualized. Absence of data about the flow inside the lesion may even lead one to hypothesize that most of the time, there is actually (almost) no flow inside the lesion, and increase in the flow – for whatever reason – may result in its rupture and hemorrhagic stroke. Interestingly, at least in our practice, cavernomas never caused symptomatic hemorrhages after diagnostic stereotactic biopsy, performed before the era of modern magnetic resonance imaging. We have not seen any report on post-biopsy bleeding in the literature, either. Furthermore, frequently identified during surgery, partial or total thrombosis inside the sinusoids indirectly indicates stagnation of blood flow. The triggering factors of flow activation, if the phenomenon exists, are not identified. On the other hand, if the blood flow inside the lesion is constant, and the aforementioned “flow interruption-re–activation” theory is thus false, the hemodynamic pressure gradient still must be very low, and one must wonder how this flow can be sufficient to cause a bleeding, with volume significantly larger than the cavernoma itself, especially in the absence of prominent feeding vessels. Or is the hemorrhage caused not only by the cavernoma, but also by pathological vessels around the lesion? Accordingly, should we pay more attention to the changes in surrounding parenchymal vessels? Undoubtedly, despite the recent advances in molecular biology of this disease, more light should be shed also on the very basic pathophysiologic characteristics of this disease.

